# The reduction of cocoa cake bitterness using natron and its effects on chocolate nutritive value

**DOI:** 10.1002/fsn3.1624

**Published:** 2020-05-07

**Authors:** Roger Ponka, Millie Dauriane Bavoua, Jean Bosco Etoa, Elie Fokou

**Affiliations:** ^1^ Department of Agriculture, Livestock and Derivated Products National Advanced School of Engineering of Maroua University of Maroua Maroua Cameroon; ^2^ Collectif des organisations pour la sécurité alimentaire et développement rural, Coordonnateur Prolinnova‐Cameroun Yaoundé Cameroon; ^3^ Department of Biochemistry Faculty of Science University of Yaoundé I Yaoundé Cameroon

**Keywords:** chocolate, Cocoa cake bitterness, natron, nutritive value

## Abstract

This study aims at finding how natron reduces the bitterness of cocoa cake and also examines its effect on chocolate nutritive value. Two hundred grams of cocoa cake was treated with different amounts of natron (0 g, 0.961 g; 1.082 g; 1.202 g; 1.322 g; and 1.442 g). Sensory analyzes were performed on each natron‐treated cake. Three ranges of chocolates (black chocolate, milk chocolate, and spread chocolate) were formulated with three cakes (cake with 0 g, 1.202 g and 1.442 g of natron). The nutritive value of the most preferred chocolates was determined by standard Association of Official Analytical Chemists (AOAC) methods. Results showed that natron significantly reduces the bitterness of cocoa cake (*p *˂ .05). The natron treatment significantly improves the taste and overall acceptability (*p *˂ .05) of all chocolate ranges. The most appreciated chocolates are those containing 1.442 g; 0 g and 1.202 g of natron, respectively, for black, milk and spread chocolate. Natron significantly decreased (*p* < .05) the phenolic composition of milk and spread chocolate as well as the moisture, lipid, ash, and energy content. Nevertheless, it significantly increases (*p* < .05) the levels of carbohydrates and fiber. The treatment with 1.202 g and 1.442 g of natron significantly increases (*p* ˂ .05) the sodium and iron content of all ranges of chocolates. For the black chocolate range, treatment with 1.442 g of natron significantly increases (*p* < .05) the levels of sodium, iron, zinc, phosphorus, magnesium, calcium, manganese and potassium. Natron treatments improve taste, overall acceptability, and sodium and iron contents of chocolate ranges.

## Graphical abstract

Natron significantly reduces cocoa cake bitterness. Natron treatments improve taste, overall acceptability, sodium, and iron contents of chocolate ranges.

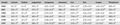

## 1 INTRODUCTION

World chocolate consumption attained 2.6 million tons, with an average of 0.97 kg/person in 2009 (Alliot, Cortin, Feige‐Muller, & Ly, 2016). Europeans, with an average of 1.87 kg/person are leading. Suisse people with 11 kg/person are first world chocolate consumers. In 2013, four million tons of chocolate were sold. This represents an increase of 32% in ten years (Alliot et al., 2016). Chocolate consumption regularly increases by 4 million tons per year.

Estimation of chocolate consumption in Africa is 4% (Puratos, 2017). This low consumption is due to habits, the product is expensive, lack of adequate material transformation and low income for producers. African countries are great world cocoa producers; their production represents 76% during 2016/2017 campaign (ICCO, 2016). Ivory Coast is the first producer, followed by Ghana, Nigeria, and Cameroon. Chocolate is one of the top class products derivate from beans cocoa companies is Suisse. With 8.4 million inhabitants, Suisse consumed 920,000 tons of chocolate products (CHOCOSSUISSE, 2017).

Cameroon produced 231,642 tons of cocoa beans during the 2016/2017 campaign. The country is planning to produce 600,000 tons in 2020 (ONCC, 2017). But international price fluctuations and the oldness of cocoa plantations are leading rural actors to develop new strategies and alternative subsistence means (Alary, 1996). Since 2006, the Ministry of Agriculture and Rural Development and the Ministry of Industry and Trade are organizing trainings for economic agents in artisanal beans transformation to limit postharvest losses and increase local income. To perform artisanal cocoa beans transformation, local chocolate consumption should be encouraged. This will contribute to rural exodus reduction and fight against poverty (Kouadio, 2011). Some local producers have started artisanal local beans transformation. But most of them did not master transformation processes. Acceptance of their final product by consumers is then raised. Bitterness contains in cocoa beans is naturally transferred in chocolate. Significant amounts of sucrose and milk are added by processors to dilute that bitter taste in manufactured chocolate final product (Hahn, 2014). Those products are expensive at local level and alter taste of the final product and reluctance among consumers particularly for artisanal chocolate.

A couple of farmers have developed a small‐scale local innovation by using natron to reduce bitterness in chocolate. They developed the innovation by linking the effect of natron in reducing bitterness for *Vernoniasp* leaves while cooking. However, adequate quantities of natron to use and its effect in nutritive value of the chocolate products are unknown. Thus, this study aims at finding how natron reduces the bitterness of cocoa cake and also examines its effect on chocolate nutritive value.

## 2 MATERIALS AND METHODS

Raw mixed cocoa beans raw materials were collected at Fegmimbang, situated at 46 km from Yaoundé the capital city of Cameroon located between latitude 2°47ꞌ– 6°5ꞌ North and between longitude 11°40ꞌ– 14° East. The main activities of the residents of this locality are agriculture and trade (CVUC, 2014). A couple of farmers in Fegmimbang carried out some postharvest operations like withdrawing cocoa beans from their pods, drying of cocoa beans under the sun, and measuring the average quantity of natron per unit of cocoa cake were done. Cocoa beans were transported into the laboratory of food sciences and metabolism of the University Yaoundé I (Cameroon) for further study.

### 2.1 Cocoa cake production

A quantity of 5 kg cocoa beans was roasted in small saucepan under low heat fire for 30 to 40 min. Once roasted, the beans were cooled and crushed. After winnowing, the crushed beans were ground using a crank. The resulting pate was then mixed with 5 L of water and homogenized until it became smooth. It was placed on low heat fire, and the mixture was constantly stirred until oil appeared. The oil was collected using ladles and kept in bottles. Cocoa cake is the remaining paste after removing the oil.

### 2.2 Treatment of cocoa cake with different amounts of natron

The determination of the amount of natron started with the evaluation of the average quantity uses by the couple of farmers through them fingers picking method. Sartorius (0.001g) balance was used to weigh natron. An average natron quantity of 1.202 g was obtained with the three following measures (1.364 g; 0.801 g; and 1.440 g). The couple of farmers use to pick 5 or 6 times with their fingers for 1000 g cocoa cake. During this experimentation, they did it five times. Then, we obtained 6.008 g natron for 1,000 g of cocoa cake. A quantity of 1.202 g natron was taken as reference for every single 200 g cocoa cake treatment. A factor of 10% variation was, respectively, used at −20%, −10%, 0%, 10%, and 20% to determine ranges of natron measures at 0.961 g; 1.082 g; 1.202 g; 1.322 g; and 1.442 g. A negative control of 0g natron measure in relation with the reference (1.202 g) was realized for effective bitterness reduction evaluation purpose. Six natron measures were applied to cocoa cake as treatments. Each of the six previous natron quantities was mixed with 200 g cocoa in a pot over a burner flame of *aifa* mark. The mixture was constantly stirred during 10 min at low heat fire.

### 2.3 Cocoa ranges formulation

#### 2.3.1 Black chocolate range

This cocoa range is made up of sugar, cocoa cake, and cocoa oil. Various ingredients (sugar and cocoa butter) were added to cocoa cake after natron treatments, and it was put in the bowl. Cocoa cake with new ingredients in the bowl was placed over a slight fire burner flame of *aifa* mark, for 5 min, and was continuously stirred. After refreshment, the chocolate that was obtained was conditioned in small pot and was stored at 4°C until analysis. The same protocol was followed for other chocolate ranges. Different formulations are presented in Table 1.

**Table 1:** Black chocolate various formulations

Chocolate sample codes	Natron (g)	Sugar (g)	Cocoa cake (g)
**SNB**	1.202	80	200
**SNH**	1.442	80	200
**SNC**	1.202	60	200
**SNA**	1.202	100	200
**SNI**	1.442	60	200
**SNN**	0	80	200
**SNO**	0	60	200
**SNM**	0	100	200
**SND**	1.202	40	200
**SNP**	0	40	200
**SNJ**	1.442	40	200
**SNR**	0	0	200
**SNF**	1.202	0	200
**SNQ**	0	20	200
**SNL**	1.442	0	200
**SNE**	1.202	20	200
**SNK**	1.442	20	200
**SNG**	1.442	100	200

Note

Attributed codes (**SNB, SNH, SNC, SNA, SNI, SNN, SNO, SNM, SND, SNP, SNJ, SNR, SNF, SNQ, SNL, SNE, SNK, and SNG)** correspond to ingredients proportion used for every single formulation.

#### 2.3.2 Milk chocolate range

The chocolate range is made with sugar, cocoa cake, milk, cocoa butter. The process is the same as black chocolate range but milk is added. Different formulations are presented in Table 2.

**Table 2:** Milk chocolate range formulations

Chocolate sample codes	Natron (g)	Sugar (g)	Milk (ml)	Cocoa cake (g)
CNJ	1.442	40	20	200
CNK	1.442	20	10	200
CNG	1.442	100	50	200
CNC	1.202	60	30	200
CNL	1.442	0	0	200
CNO	0	60	30	200
CNA	1.202	100	50	200
CNI	1.442	60	30	200
CNB	1.202	80	40	200
CNN	0	80	40	200
CNQ	0	20	10	200
CNM	0	100	50	200
CNR	0	0	0	200
CNE	1.202	20	10	200
CNH	1.442	80	40	200
CNF	1.202	0	0	200
CND	1.202	40	20	200
CNP	0	40	20	200

Note

Attributed codes (CNJ, CNK, CNG, CNC, CNL, CNO, CAN, CNI, CNB, CNN, CNQ, CNM, CNR, CNE, CNH, CNF, CND, and CNP) correspond to ingredients proportion used for every single formulation

#### 2.3.3 Spreading chocolate range

This chocolate range is made by associating sugar, milk, cocoa butter, groundnuts paste and cocoa cake. Methods and conditions of preparation are the same as previous ones. Different formulations are presented in Table 3.

**Table 3:** Spreading chocolate various formulations

Chocolate sample codes	Natron (g)	Groundnuts paste (g)	Sugar (g)	Milk (ml)	Cocoa cake (g)
**CNH’**	1.442	48	80	50	200
**CNC’**	1.202	36	60	30	200
**CNB’**	1.202	48	80	40	200
**CNR’**	0	0	0	0	200
**CNM’**	0	60	100	50	200
**CNE’**	1.202	12	20	10	200
**CNI’**	1.202	36	60	30	200
**CNL’**	1.442	0	0	0	200
**CNG’**	1.442	60	100	50	200
**CNP’**	0	24	40	20	200
**CNJ’**	1.442	24	40	20	200
**CND’**	1.202	24	40	20	200
**CNA’**	1.202	60	100	50	200
**CNF’**	1.202	0	0	0	200
**CNN’**	0	48	80	40	200
**CNK’**	1.442	12	20	10	200
**CNO’**	0	36	60	30	200
**CNQ’**	0	12	20	10	200

Note

Attributed codes (**CNH’, CNC’, CNB’, CNR’, CNM’, CNE’ CNI’, CNL’, CNG’, CNP’, CNJ’, CND’, CNA’, CNF’, CNN’, CNK’, CNO’, and CNQ’)** correspond to ingredients proportion used for every single formulation

#### 2.3.4 Sensory evaluation of cocoa cakes and chocolate ranges

The products were subjected to a sensory test using 35 panelists. Each attribute was scored based on its intensity scaled on a 9‐point hedonic scale (1 = extremely bitter; 2 = very bitter; 3 = moderately bitter; 4 = slightly bitter; 5 = not bitter, not sweet; 6 = slightly sweet; 7 = moderately sweet; 8 = very sweet; 9 = extremely sweet) for taste of **cocoa cakes.** (1 = disliked extremely; 2 = disliked very much; 3 = disliked moderately; 4 = disliked slightly; 5 = neither liked or disliked; 6 = liked slightly; 7 = like moderately; 8 = liked very much; 9 = liked very extremely) for color, taste, texture, and overall acceptability for taste **of chocolate ranges.**

### 2.4 Proximate composition

The moisture content, ash, fat, protein, and fiber of all the samples were determined according to the method of AOAC (2000). All samples were analyzed in triplicate. Total carbohydrate content was calculated by difference.

### 2.5 Mineral determination

The mineral content (calcium, magnesium, sodium, potassium, iron copper, zinc, and manganese) was determined according to the standard methods of the Association of Official Analytical Chemists AOAC (2005), using an atomic absorption spectrometer. The sample was ashed at 550°C and the ash boiled with 10 ml of 20% HCl in a beaker and then filtered into a 100 ml standard flask. Phosphorus was determined colorimetrically using the vanado molybdate method (AOAC, 1999). All samples were analyzed in triplicate.

### 2.6 Total polyphenol and flavonoid contents

Total polyphenol and Flavonoid contents were determined colorimetrically according to the methods of Ribereau‐Gayon, Peynaud, and sudraud (1972) and Ayoola et al. (2008), respectively.

### 2.7 Statistical analysis

Data were presented as means ± standard deviation (*SD*).Values were statistically analyzed by one‐way analysis of variance (ANOVA test) using IBM SPSS Statistics version 20.0.1. Software package, Armonk., New York. USA. Differences were considered significant at *p* < .05 using Duncan Multiple Range test.

## 3 RESULTS AND DISCUSSION

### 3.1 Effect of natron on cocoa cake bitterness

Table 4 shows the rank and degree of bitterness of the various natron‐treated cocoa cakes. According to the classification of the consumers, the least bitter cake is the one with 1.442g of natron (PN5) followed by the cake PN4, PN3, PN2, PN1, and PN0 (PN5 < PN4 <PN3 < PN2 <PN1 < PN0). Bitterness decreased significantly (*p* < .05) with increasing amounts of natron. These results are in accordance with those of Wissgott (1987) which showed that, after alkalization, the cocoa mass is less bitter.

**Table 4:** Rank of cocoa cakes processed with natron

Cocoa cake	Degree of bitterness decreasing cocoa cakes treated with natron Rank of cocoa cakes	Rank of cocoa cakes
**PN0**	2.47 ± 1.06^a^	6
**PN1**	2.72 ± 0.88^ab^	5
**PN2**	3.47 ± 1.056^cd^	4
**PN3**	3.25 ± 1.13^bc^	3
**PN4**	3.58 ± 1.18^cd^	2
**PN5**	4.14 ± 1.09^d^	1

Note

Mean values with different superscript letters are significantly different (*p* < .05).

**PN0**: cocoa cake treated with 0 g natron; **PN1**: cocoa cake treated with 0.961g natron; **PN2**: cocoa cake treated with 1.082g natron; **PN3**: cocoa cake treated with 1.202g natron; **PN4**: cocoa cake treated with 1.322g natron; **PN5**: cocoa cake treated with 1.442g natron.

### 3.2 Effects of natron on the acceptability of chocolate

Table 5 shows the sensory evaluation of black chocolate samples. The value ranged between 5 and 6.49 for color, taste (2.00–7.26), texture (3.89 –6.83), and overall acceptability (3.00–7.11). Sample SNG has the highest score ranging from 6.49 color, taste (7.26), texture (6.83), and overall acceptability (7.11). This could be due to the effect of natron. Through bitterness reduction with natron cocoa cake pretreatment, the final taste of product is improved and is more accepted. These results are correlated with those of Wissgott (1987) which shows that alkalinization improves the taste of chocolate products.

**Table 5:** Sensory evaluation of black chocolate samples

Black chocolate samples	Color	Taste	Texture	Overall acceptability
**SNB**	5.14 ± 1.83^a^	4.89 ± 1.76^def^	4.60 ± 1.67^ab^	4.69 ± 1.75^abc^
**SNH**	5.23 ± 2.13^a^	5.26 ± 1.95^def^	4.89 ± 1.94^ab^	5.09 ± 1.88^bcd^
**SNC**	5.20 ± 1.92^a^	4.89 ± 1.35^def^	4.86 ± 1.88^ab^	4.97 ± 1.58^bcd^
**SNA**	5.34 ± 2.18^a^	5.77 ± 1.37^ef^	5.00 ± 1.83^ab^	5.66 ± 1.51^cde^
**SNI**	5.94 ± 1.81^a^	5.46 ± 1.50^ef^	5.80 ± 1.69^bc^	5.77 ± 1.65^cde^
**SNN**	5.00 ± 1.94^a^	4.57 ± 2.06^cdef^	4.57 ± 1.74^ab^	4.51 ± 1.88^abc^
**SNO**	5.89 ± 1.68^a^	4.91 ± 1.44^def^	5.17 ± 1.64^ab^	5.17 ± 1.49^bcd^
**SNM**	5.91 ± 1.65^a^	5.66 ± 1.49^ef^	5.80 ± 1.57^bc^	6.03 ± 1.32^cde^
**SND**	5.77 ± 1.73^a^	4.71 ± 1.20^cdef^	4.91 ± 1.74^ab^	4.97 ± 1.29^bcd^
**SNP**	6.34 ± 1.60^a^	5.06 ± 1.61^def^	5.29 ± 1.47^ab^	6.57 ± 6.85^de^
**SNJ**	5.06 ± 1.71^a^	4.17 ± 1.49^cd^	4.29 ± 1.90^a^	4.34 ± 1.41^abc^
**SNR**	5.37 ± 2.57^a^	2.00 ± 1.39^a^	4.06 ± 2.18^a^	3.06 ± 1.83^a^
**SNF**	5.57 ± 2.29^a^	2.20 ± 1.32^a^	4.14 ± 2.10^a^	3.00 ± 1.52^a^
**SNQ**	5.11 ± 1.79^a^	3.54 ± 1.22^bc^	4.03 ± 1.56^a^	3.69 ± 1.39^ab^
**SNL**	5.74 ± 1.84^a^	2.83 ± 1.56^ab^	3.89 ± 1.88^a^	3.43 ± 1.63^ab^
**SNE**	5.51 ± 1.67^a^	4.37 ± 1.56^cde^	4.71 ± 2.02^ab^	4.57 ± 1.60^abc^
**SNK**	5.74 ± 1.69^a^	4.60 ± 1.48^def^	4.77 ± 1.78^ab^	4.66 ± 1.43^abc^
**SNG**	6.49 ± 2.10^a^	7.26 ± 1.52^f^	6.83 ± 1.62^c^	7.11 ± 1.53^e^

Note

Mean values in the same column with different superscript letters are significantly different (*p* < .05).

Attributed codes (**SNB, SNH, SNC, SNA, SNI, SNN, SNO, SNM, SND, SNP, SNJ, SNR, SNF, SNQ, SNL, SNE, SNK, and SNG)** correspond to ingredients proportion used for every single formulation for Black chocolate samples presented in Table 1.

Table 6 shows the sensory evaluation of milk chocolate samples. The value ranged between 4.06 and 6.51 for color, taste (2.26–6.80), texture (2.94–6.40), and overall acceptability (2.37–6.89). Sample CNM has the highest score ranging from 6.51 color, taste (6.80), texture (6.40), and overall acceptability (6.89). Addition of milk strongly affects natron acceptance or rejection of chocolate. Bitter reduction due to natron treatment and milk presence seems to provoke over chocolate sweetness and rejection by consumers.

**Table 6:** Sensory evaluation of milk chocolate samples

Chocolate samples	Color	Taste	Texture	Overall acceptability
CNJ	4.91 ± 1.74^ab^	4.46 ± 1.90^bcd^	4.49 ± 1.84^bcde^	4.80 ± 1.64^cd^
CNK	4.83 ± 2.05^a^	3.31 ± 2.04^ab^	3.37 ± 1.83^abc^	3.91 ± 1.79^bc^
CNG	5.57 ± 2.33^ab^	6.60 ± 1.42^e^	6.17 ± 1.89^fg^	6.60 ± 1.44^e^
CNC	6.03 ± 1.74^b^	5.57 ± 2.31^cde^	5.43 ± 1.96^defg^	5.66 ± 1.98^de^
CNL	4.94 ± 2.75^ab^	2.26 ± 1.79a	2.94 ± 2.16^a^	2.37 ± 1.50^a^
CNO	6.46 ± 1.70^b^	5.74 ± 1.82^cde^	6.00 ± 1.48^efg^	5.91 ± 1.38^de^
CNA	5.20 ± 2.11^ab^	6.31 ± 1.45^e^	5.74 ± 1.67^efg^	6.20 ± 1.26^e^
CNI	5.34 ± 1.75^ab^	5.80 ± 1.61^cde^	5.80 ± 1.49^efg^	5.94 ± 1.37^de^
CNB	6.26 ± 1.46^b^	6.46 ± 1.36^e^	6.03 ± 1.54^fg^	6.31 ± 1.18^e^
CNN	6.49 ± 1.52^b^	6.23 ± 1.91^e^	5.89 ± 1.83^efg^	6.29 ± 1.50^e^
CNQ	5.20 ± 2.60^ab^	3.40 ± 2.42^ab^	4.11 ± 2.34^abcd^	4.00 ± 2.26^bc^
CNM	6.51 ± 1.73^b^	6.80 ± 1.39^e^	6.40 ± 1.44^g^	6.89 ± 1.32^e^
CNR	4.14 ± 2.65^a^	2.26 ± 1.88^a^	3.57 ± 2.40^abc^	2.80 ± 2.07^ab^
CNE	5.03 ± 1.93^ab^	4.43 ± 1.99^bc^	4.66 ± 1.83^cdef^	4.60 ± 1.85^cd^
CNH	5.23 ± 2.18^ab^	6.63 ± 1.44^e^	5.83 ± 1.54^efg^	6.51 ± 1.54^e^
CNF	4.06 ± 2.39^a^	2.34 ± 1.80^a^	3.03 ± 2.14^ab^	2.86 ± 1.73^ab^
CND	5.66 ± 1.80^ab^	5.69 ± 1.86^cde^	5.80 ± 1.84^efg^	5.86 ± 1.59^de^
CNP	6.37 ± 1.20^b^	5.94 ± 1.51^de^	6.09 ± 1.36^fg^	6.29 ± 1.18^e^

Note

Mean values in the same column with different superscript letters are significantly different (*p* < .05).

Attributed codes (CNJ, CNK, CNG, CNC, CNL, CNO, CAN, CNI, CNB, CNN, CNQ, CNM, CNR, CNE, CNH, CNF, CND, and CNP) correspond to ingredients proportion used for every single formulation for milk chocolate samples presented in Table 2.

Table 7 shows the sensory evaluation of spreading chocolate samples. The value ranged between 5.21 and 6.61 for color, taste (2.58–6.95), texture (3.32 –6.74), and overall acceptability (2.82–7.11). Sample **CNA’** has the highest score ranging from 6.51 color, taste (6.95), texture (6.74), and overall acceptability (7.11).

**Table 7:** Sensory evaluation of spreading chocolate samples

Chocolate samples	Color	Taste	Texture	Overall acceptability
**CNH’**	5.79 ± 1.66^a^	5.11 ± 1.98^bcde^	4.92 ± 1.94^bcde^	5.53 ± 2.05^bc^
**CNC’**	5.97 ± 1.80^a^	5.50 ± 2.01^bcdef^	5.74 ± 1.62^def^	5.82 ± 1.69^bcd^
**CNB’**	6.13 ± 1.79^a^	5.97 ± 1.94^def^	5.47 ± 1.61^cdef^	6.05 ± 1.77^bcd^
**CNR’**	5.63 ± 2.64^a^	2.58 ± 2.20^a^	3.32 ± 2.53^a^	2.82 ± 2.13^a^
**CNM’**	6.47 ± 1.67^a^	6.32 ± 1.53^ef^	6.50 ± 1.81^f^	6.63 ± 1.68^cd^
**CNE’**	5.55 ± 1.75^a^	4.37 ± 2.15^b^	4.68 ± 1.85^abcd^	4.84 ± 1.84^b^
**CNI’**	6.37 ± 1.65^a^	6.29 ± 1.51^ef^	6.08 ± 1.71^ef^	6.47 ± 1.50^cd^
**CNL’**	5.34 ± 2.46^a^	2.84 ± 2.31^a^	4.00 ± 2.60^abc^	3.18 ± 2.40^a^
**CNG’**	6.08 ± 1.82^a^	6.39 ± 1.46^ef^	5.89 ± 1.66^def^	6.47 ± 1.50^cd^
**CNP’**	5.76 ± 1.90^a^	5.18 ± 1.83^bcde^	5.68 ± 1.80^def^	5.42 ± 1.77^bc^
**CNJ’**	5.66 ± 2.12^a^	5.87 ± 1.77^cdef^	5.63 ± 1.68^def^	5.95 ± 1.69^bcd^
**CND’**	6.24 ± 1.62^a^	6.00 ± 1.47^def^	5.97 ± 1.42^def^	6.03 ± 1.53^bcd^
**CNA’**	6.61 ± 1.61^a^	6.95 ± 1.58^f^	6.74 ± 1.59^f^	7.11 ± 1.72^d^
**CNF’**	5.21 ± 2.57^a^	2.66 ± 2.18^a^	3.71 ± 2.24^ab^	2.82 ± 2.08^a^
**CNN’**	6.50 ± 1.67^a^	6.32 ± 1.51^ef^	6.53 ± 1.75^f^	6.45 ± 1.55^cd^
**CNK’**	5.66 ± 1.60^a^	4.53 ± 1.75^bcd^	4.82 ± 1.84^abcd^	4.79 ± 1.85^b^
**CNO’**	6.58 ± 1.45^a^	6.24 ± 1.62^ef^	6.37 ± 1.57^ef^	6.50 ± 1.56^cd^
**CNQ’**	5.63 ± 1.92^a^	4.47 ± 2.09^bc^	5.26 ± 1.99^cdef^	4.89 ± 1.96^b^

Note

Mean values in the same column with different superscript letters are significantly different (*p* < .05).

Attributed codes (**CNH’, CNC’, CNB’, CNR’, CNM’, CNE’ CNI’, CNL’, CNG’, CNP’, CNJ’, CND’, CNA’, CNF’, CNN’, CNK’, CNO’, and CNQ’)** correspond to ingredients proportion used for every single formulation of spreading chocolate samples presented in Table 3.

### 3.3 Effects of natron on the total polyphenols and flavonoids in different most appreciated chocolate samples

Table 8 shows the effects of natron on the total polyphenols and flavonoids in different most appreciated chocolate samples. The total polyphenols of the samples ranged from 8.05 for CNM’ to 11.28 g/100 g DM for SNG and were significantly different (*p* < .05). Flavonoids varied from 1.83 for CNA’ to 3.74 g/100g DM for SNG. The contents of total polyphenols in black chocolates (SNM and SNG) are 10.31 and 11.28 g/100 g DM, respectively. Those of milk chocolates (CNM and CNG) being, respectively, 10.11 and 9.30 g/ 100 g DM and that of spreads chocolate (CNM ' and CNA') being, respectively, 8.05 and 8.59 g/ 100 g MS. The contents of total polyphenols in black chocolates were higher than those in milk and spread chocolate. The same is true for the flavonoid contents which were, respectively, 3.74 and 3.19 g/ 100 g DM for black chocolates (SNG and SNM); 2.67 and 2.98 g/ 100 g DM for milk chocolates; and, respectively, 1.83 and 2.33 g/ 100 g DM for chocolate spreads (CNM 'and CNA'). These results show that the flavonoid content decreases according to the quantity of natron and the different ranges of chocolates. This could be due to the antioxidant properties. These results are in agreement with those of Moser (2015), who showed that the alkalinization of chocolate decreases the quantity of flavonoids. They are also in harmony with those of Wollgast and Aklam (2000), who obtained a flavonoid content of 6% for black chocolate and 2% for milk chocolate. The natron treatment has a weak influence on the flavonoid content of the different chocolate ranges compared to the polyphenol content. Natron treatment reduces half of polyphenol contents contained in cocoa beans (23.43 g/100 g). Polyphenol contents of black chocolate samples increase depending on natron treatment. This result is in disagreement with that of Wissgott (1987) who shows that the treatment of cocoa cake with an alkaline agent favors the treatment of polyphenols, responsible in large part for the red color, bitterness, chocolate taste, and the solubility of cocoa pigments.

**Table 8:** Total polyphenols and flavonoids in different most appreciated chocolate samples (g/100g DM)

chocolate samples	Total Polyphenols	Flavonoids
**SNM**	10.31 ± 0.14^bc^	3.19 ± 0.07^d^
**SNG**	11.28 ± 0.34^c^	3.74 ± 0.17^e^
**CNM**	10.11 ± 1.61^bc^	2.67 ± 0.18^bc^
**CNG**	9.30 ± 0.10^ab^	2.98 ± 0.11^cd^
**CNM’**	8.05 ± 0.52 ^a^	2.33 ± 0.248^b^
**CNA’**	8.57 ± 0.11^ab^	1.83 ± 0.054^a^

Note

Mean values in the same column with different superscript letters are significantly different (*p* < .05).

**SNM** (Control): black chocolate sample with 100 g sugar, 20 ml cocoa butter, 200 g cocoa cake, 0 g natron. **SNG**: black chocolate with 100 g sugar, 200 ml cocoa butter, 200 g cocoa cake, and 1.442 g natron. **CNM** (Control): Milk chocolate 100 sugars, 20 ml cocoa butter, 50 ml milk, 200 g cocoa cake, and 0 g natron.**CNG**: milk chocolate with 100 g sugar, 20 ml cocoa butter, 50 ml milk, 200 g cocoa cake, and 1.442 g natron. **CNM’** (Control): spreading chocolate with 100 g sugar; 20 ml cocoa butter; 50 ml milk, 200 g cocoa cake, and 0 g natron. **CNA’:** spreading chocolate with 100 g sugar, 20 ml cocoa butter, 50 ml milk, 200 g cocoa cake, and 1.202 g natron.

On the other hand, the polyphenol content of milk chocolate decreases from the control to the chocolate treated with natron. It is the same for the flavonoid contents which increase from the control chocolate to the chocolate treated with natron, respectively, for the black chocolate and milk chocolate. The reverse phenomenon is observed for chocolate spread chocolate where the flavonoid content decreases from control chocolate to chocolate treated with natron. This could be due to the hydrolysis caused by natron. Indeed, the treatment of flavonoids by hydrolysis with natron would have caused their number to drop in the final product because according to Noble and Fisher (1994), the bitterness is linked to the presence of the monomers and polymers of flavonol‐3ol catechin and epicathechin.

### 3.4 Effects of natron on the proximate composition of the different most appreciated chocolate samples

The proximate composition of the different chocolate samples is presented in Table 9. The moisture content in the sample ranged from 23.10 for SNG to 39.29 g/100 gMF for CNM and was significantly different (*p* < .05). The low moisture contents are due to the absorbent properties of natron. When used in powder form, natron traps the water molecules present in chocolate. The moisture contents of the chocolates decrease with the natron treatment.

**Table 9:** Proximate composition of the different chocolate samples

Samples	Moisture (g/100 gFM)	Ash (g/100 g DM)	Fat (g/100 g DM)	Protein (g/100 g DM)	Fiber (g/100 g DM)	Carbohydrate (g/100 g DM)	Energy (Kcal/100 g DM)
**SNM**	25.51 ± 0.15^b^	1.07 ± 0.02^ab^	43.36 ± 0.21^e^	9.98 ± 0.06^e^	3.65 ± 2.03^a^	43.12 ± 0.04^b^	601.90 ± 2.27^e^
**SNG**	23.10 ± 0.53^a^	1.03 ± 0.09^ab^	35.69 ± 0.55^d^	2.93 ± 0.16^c^	9.70 ± 0.10^c^	50.67 ± 0.52^d^	536.69 ± 3.98^c^
**CNM**	35.29 ± 0.03^e^	0.76 ± 0.10 ^a^	32.86 ± 0.70^c^	5.38 ± 0.06^d^	5.4 ± 0.19^b^	55.96 ± 1.20^e^	538.88 ± 2.70^c^
**CNG**	28.31 ± 0.36^c^	1.08 ± 0.15^ab^	23.86 ± 0.48^a^	2.45 ± 0.03^b^	11.31 ± 0.35^d^	61.64 ± 1.16^f^	469.61 ± 0.46^a^
**CNM'**	31.19 ± 0.31^d^	1.35 ± 0.25^b^	44.34 ± 0.30^f^	2.91 ± 0.19^c^	5.21 ± 0.09^b^	46.20 ± 0.15^c^	595.44 ± 2.84^d^
**CNA'**	25.39 ± 0.05^b^	1.38 ± 0.43^b^	27.94 ± 0.52^b^	0.82 ± 0.06^a^	4.94 ± 0.02^ab^	64.88 ± 0.18^g^	515.83 ± 4.89^b^

Note

Mean values in the same column with different superscript letters are significantly different (*p* < .05).

**SNM** (Control): black chocolate sample with 100 g sugar, 20 ml cocoa butter, 200 g cocoa cake, 0 g natron. **SNG**: black chocolate with 100 g sugar, 200 ml cocoa butter, 200 g cocoa cake, and 1.442 g natron. **CNM** (Control): Milk chocolate 100 sugars, 20 ml cocoa butter, 50 ml milk, 200 g cocoa cake, and 0 g natron. **CNG**: milk chocolate with 100 g sugar, 20 ml cocoa butter, 50 ml milk, 200 g cocoa cake, and 1.442 g natron. **CNM’** (Control): spreading chocolate with 100 g sugar; 20 ml cocoa butter; 50 ml milk, 200 g cocoa cake, and 0 g natron. **CNA’:** spreading chocolate with 100 g sugar, 20 ml cocoa butter, 50 ml milk, 200 g cocoa cake, and 1.202 g natron.

The ash content in the samples was in the range of 0.76 (CNM) to 1.38 g/100g DM (CNA') and was significantly different (*p* < .05). The ash content increases with increasing amounts of natron. These results are correlated with those of Balla and Baragé (2006), who found higher concentrations with increasing amounts of natron.

The fat content in the sample ranged from 23.86 for CNG to 44.34 g/100g DM for CNM' and was significantly different (*p* < .05). Natron treatment considerably reduces the lipid content of chocolates. This reduction could be due to the interaction between natron and free fatty acids, as explained by Nikita (2013). The alkalization of chocolate releases cocoa butter and the nonfatty components are in the emulsion phase (Bill, 2003).

Protein content in the sample varied from 0.82 for CNA' to 9.98 g/100 g DM for SNM and was significantly different (*p* < .05). It is found that the protein contents decrease from the control chocolates to the chocolates treated with natron. However, the high amount of natron (1.442g) considerably reduces the protein content compared to the low amount of natron (1.202g). This could be explained by the precipitation of proteins caused by alkalization due to natron which is combined with the effect of temperature. Because according to Bill (2003), proteins undergo irreversible chemical changes due to heat and hydration during alkalization.

The fiber content in the sample was in the range of 3.65 to 9.70 g/100g DM. The lowest fiber content was obtained from SNM while the highest was obtained from SNG. The values of fiber content were significantly different (*p* < .05). The fiber content of black chocolates and milk chocolates is increasing by comparison with chocolates treated with natron. However, fiber content of spreads chocolate decreases depending on the treatment. These results could be explained by the different composition of the chocolate ranges. Fiber decreases the absorption of fat, blood cholesterol, and triglycerides and helps prevent cardiovascular disease. It also has the advantage of slowing down the absorption of carbohydrates and therefore slowing the rise of blood sugar, which is essential in the prevention of type 2 diabetes (Daverio, 2005).

Carbohydrate content in the sample ranged between 43.12 for SNM to 64.88 g/100 g DM for CNA' and was significantly different (*p* < .05). The natron treatment considerably increases the carbohydrate content of the chocolates. This could be due to the increase in the content of complex sugars (especially fibers) induced by the addition of natron. According to Bill (2003), starches undergo irreversible chemical changes due to heat and hydration during alkalization, making them unavailable in the final product.

The energy content in the sample varied from 469.61 for CNG from 601.90 Kcal/100 g DM for SNM and was significantly different (*p* < .05). The energy value of black and milk chocolates increases following the natron treatment. The small amounts of natron (1.202 g) reduce the energy value of chocolates in contrast to large amounts of natron (1.442 g). This could be due to the difference in the ranges of chocolates.

### 3.5 Effects of natron on the mineral composition of the different most appreciated chocolate samples

Table 10 presents mineral composition of the samples. The calcium content in the sample varied from 76 for SNM from 128 mg/100 g DM for CNM and was significantly different (*p* < .05). The calcium content increases according to the chocolate ranges and varies very little according to natron treatments. These results could be due to the presence of milk whose richness in calcium increases that of chocolates. Calcium in addition to its role on bone mass is also involved in the mechanism of muscle contraction. It plays a role in the cascade of blood clotting and in the metabolism of many hormones (Degossely, 2000). EFSA (2015) recommends that people aged 19 to 50 and over have a daily calcium intake of 1,200 mg per day. Since natron treatment increases the calcium content, as well as the addition of milk, the different ranges of chocolates treated with natron would be beneficial for strengthening bones and reducing calcium deficiencies.

**Table 10:** Mineral composition of the samples (mg/100 g DM)

Sample	Calcium	Sodium	magnesium	manganese	potassium	Iron	Zinc	Copper	Phosphorus
**SNM**	76 ± 5^a^	6 ± 3^a^	159 ± 5^a^	2.01 ± 0.21^a^	472 ± 5^a^	2.81 ± 0.2^a^	2.33 ± 0.2^a^	1.61 ± 0.1^ab^	240 ± 5^a^
**SNG**	102 ± 5^b^	171 ± 5^d^	174 ± 5^b^	2.24 ± 0.2^ab^	508 ± 5^b^	8.52 ± 1^d^	2.5 ± 0.2^ab^	1.56 ± 0.1^ab^	250 ± 5^b^
**CNM**	128 ± 5^d^	19 ± 5^b^	211 ± 5^d^	2.93 ± 0.2^c^	571 ± 5^d^	4.07 ± 1^ab^	3.61 ± 0.2^c^	2.27 ± 0.2^c^	350 ± 5^e^
**CNG**	119 ± 5^c^	176 ± 5^d^	183 ± 5^b^	2.46 ± 0.2^b^	524 ± 5^c^	7.36 ± 1^cd^	2.71 ± 0.2^b^	1.85 ± 0.1^b^	280 ± 5^c^
**CNM'**	102 ± 5^b^	15 ± 5^b^	217 ± 5^d^	2.38 ± 0.2^b^	731 ± 5^f^	3.75 ± 1^ab^	3.37 ± 0.2 ^c^	1.67 ± 0.1^ab^	390 ± 5^f^
**CNA'**	102 ± 5^b^	109 ± 5^c^	188 ± 5^c^	2.14 ± 0.12^ab^	664 ± 5^e^	5.42 ± 1^bc^	2.74 ± 0.2^b^	1.5 ± 0.1^a^	340 ± 5^d^

Note

Mean values in the same column with different superscript letters are significantly different (*p* < .05).

**SNM** (Control): black chocolate sample with 100 g sugar, 20 ml cocoa butter, 200 g cocoa cake, 0 g natron. **SNG**: black chocolate with 100 g sugar, 200 ml cocoa butter, 200 g cocoa cake, and 1.442 g natron. **CNM** (Control): Milk chocolate 100 sugars, 20 ml cocoa butter, 50 ml milk, 200 g cocoa cake, and 0 g natron. **CNG**: milk chocolate with 100 g sugar, 20 ml cocoa butter, 50 ml milk, 200 g cocoa cake, and 1.442 g natron. **CNM’** (Control): spreading chocolate with 100 g sugar; 20 ml cocoa butter; 50 ml milk, 200 g cocoa cake, and 0 g natron. **CNA’:** spreading chocolate with 100 g sugar, 20 ml cocoa butter, 50 ml milk, 200 g cocoa cake, and 1.202 g natron.

The Sodium content in the sample ranged from 6 for SNM to 176 mg/100 g DM for CNG and was significantly different (*p* < .05). The sodium content of the different ranges of chocolates increases according to natron treatments (regardless of the amount of natron) and according to the incorporation of different ingredients. This increase could be due to the composition of natron. It is a mixture of salts whose main constituents are sodium carbonate (Na2CO3 and NaHCO3); sodium sulfate (Na2SO4); and sodium chloride (NaCl) (Bourgueil, 1992). It is this richness in sodium that would explain the increase in sodium content in samples of chocolates treated with natron. These results are in agreement with those of Balla and Baragé (2006), who noted an increase in sodium content after natron treatment.

A significant difference was also observed in the distribution of magnesium, manganese, and potassium in the chocolate (*p* < .05). Natron treatment increases the magnesium, manganese, and potassium content of black chocolate (SNG) treated with 1.442g of natron. On the other hand, this content decreases for the ranges of milk chocolate and spreads, treated, respectively, with 1.202 g and 1.442 g of natron. These results could be due to the reaction of complexions between natron and the minerals coming from the incorporated ingredients (peanut milk paste) during the formulation. The daily consumption of manganese is 2 mg per day (AFSSA, 2001). Manganese plays a role in the formation of hormones including the synthesis of sex hormones, and the functioning of the nervous system. The consumption of natron‐treated chocolates (CNA’, SNG, and CNG) will largely cover consumers' magnesium requirements.

The iron content in the sample ranged from 2.81 for SNM to 8.52 mg/100g DM for CNG and was significantly different (*p* < .05). Natron treatment increases the iron content of the different chocolate ranges; this regardless of the amount of natron used for treatment. Iron is used in the living organism mainly to transport oxygen, or to catalyze electron transfer reactions, nitrogen fixation for the synthesis of deoxyribonucleic acid (Beaumont, 2018). The iron richness of natron‐treated chocolates (SNG, CNG, and CNA’) is an important source indicated in individuals suffering from heart function pathologies linked to iron deficiency.

The zinc content in the sample ranged from 2.33 for SNM to 3.61 mg/100g DM for CNM and was significantly different (*p* < .05). Zinc plays a catalytic and metabolic role by making the active site of nearly 300 enzymes. It also participates in the storage and release of insulin, the secretion of digestive enzymes or the secretion of acid by the parietal cells of the stomach. Finally, it has regulatory roles in terms of gene expression and intracellular signaling and it is an effective antioxidant (EFSA, 2014). In adults, the recommended daily intake of zinc is 11 mg for men and 8 mg for women, with an increase during pregnancy and lactation.

The copper content in the sample ranged from 1.5 for CNA' to 2.27 mg/100g DM for CNM and was significantly different (*p* < .05). The natron treatment reduces the copper content of the chocolates despite natron's composition of copper oxide. These results could be due to oxidation of copper caused by the presence of oxygen and temperature. Copper participates in the activity of many enzymes and chemical reactions. It is involved in the oxidation of glucose and is essential for the proper functioning of the myocardium (muscle of the heart). Copper controls bone mineralization and the quality of cartilage and plays a role in the regulation of neurotransmitters, by regulating mood, sleep, memory, and attention. Copper is also involved in the immune process and iron metabolism (Davis, Mertz, & et Copper, 1987). Consumption of chocolates treated with natron will allow consumers to fill their daily copper intake which is 1–2 mg per day (EFSA, 2015).

The phosphorus content in the sample ranged from 240 for SNM to 390mg/100g DM for CNM' and was significantly different (*p* < .05). The natron treatment increases the phosphorus content of black chocolate treated with 1.442 g of natron. On the other hand, we notice the opposite effect for the ranges of milk and natron chocolates treated, respectively, with 1.442 and 1.202 g of natron. This could be due to the binding of phosphorus with other constituents present in the natron; which would explain its low content after natron treatment. In the organism, phosphorus allows bone fixation of calcium by reducing its urinary excretion and enters the mechanism of energy storage and release (EFSA, 2014).

## 4 CONCLUSION

From this study, it appears that natron significantly reduces cocoa cake bitterness. Natron treatment significantly improves the taste and general acceptability of all chocolate ranges. The most appreciated chocolates were those containing 1.442g; 0 g; and 1.202 g of natron, respectively, for black, milk, and spread chocolate. Natron significantly reduces the phenolic composition of milk and spread chocolates as well as the moisture, lipid, ash, and energy content. Nevertheless, it significantly increases the carbohydrate and fiber contents. Treatment with 1.202 g and 1.442 g of natron significantly increases the sodium and iron content of all ranges of chocolates. For the black chocolate range, treatment with 1.442 g of natron significantly increases the contents of sodium, iron, zinc, phosphorus, magnesium, calcium, manganese, and potassium. Natron treatments improve taste, overall acceptability, sodium, and iron contents of chocolate ranges.

## CONFLICT OF INTEREST

The authors declare that they do not have any conflict of interest.
